# DOG1 Immunohistochemical Expression in Normal and Neoplastic Canine Tissues: Is It Only a Marker for GISTs?

**DOI:** 10.3390/ani16020295

**Published:** 2026-01-18

**Authors:** Maria Morini, Francesca Gobbo, Luciana Mandrioli, Giuliano Bettini

**Affiliations:** Department of Veterinary Medical Sciences, University of Bologna, Via Tolara di Sopra 50, Ozzano dell’Emilia, 40064 Bologna, Italy; francesca.gobbo3@unibo.it (F.G.); mandrioli.luciana@unibo.it (L.M.); giuliano.bettini@unibo.it (G.B.)

**Keywords:** gastrointestinal stromal tumor, ANO1, TMEM16A, dog, immunohistochemistry

## Abstract

DOG1 is a transmembrane protein highly expressed in human GISTs. In dogs, DOG1 has been studied to a limited extent in GISTs, where its diagnostic value is considered comparable to KIT, while its expression in other canine tissues remains uncharacterized. The aim of this study is to assess the expression of DOG1 in canine normal and neoplastic tissues, with particular emphasis on a large cohort of GISTs to verify their diagnostic role in comparison with KIT. To achieve this, we analyzed a total of 143 FFPE samples of normal (*n* = 55) and neoplastic canine tissues (*n* = 88) by immunohistochemistry. DOG1 was strongly expressed in several cell types, with distribution and intensity patterns that partially overlap those reported in humans. In canine GISTs, DOG1 showed strong and widespread reactivity with a sensitivity slightly inferior to KIT (92% vs. 96%); however, one intestinal mesenchymal tumor KIT-negative/DOG1-positive highlights the importance of combining both markers to maximize the correct diagnosis. Moderate to strong immunohistochemistry for DOG1 has been found in several other tumors, sometimes comparable to that of GISTs. This suggests that DOG1 should not be associated exclusively with GISTs and may provide a basis for further investigation into the role of DOG1 in canine malignancy.

## Simple Summary

A wide range of normal and neoplastic canine tissue samples were analyzed by immunohistochemistry in order to characterize the expression, distribution, and cellular localization of DOG1, the diagnostic marker for gastrointestinal stromal tumors (GISTs) in human pathology. Strong DOG1 immunoexpression was observed in several normal cell types other than interstitial cells of Cajal, including ovarian, testicular, thyroid, renal, salivary gland, and perivascular cells, partially similar to human expression patterns. Among canine tumors, DOG1 exhibited in GISTs a high diagnostic sensitivity, slightly lower than KIT, with results that highlight the diagnostic benefit of combining both markers. Notably, several spindle cell tumors and a fair number of carcinomas showed DOG1 levels comparable to those of GISTs, provided that DOG1 expression extends beyond GISTs and could have potentially significant implications also for additional canine malignancies.

## 1. Introduction

DOG1 (Discovered on GIST1), also known as Anoctamin1 (ANO1) or TMEM16A, is a transmembrane protein, and it is one of the major components of the calcium-activated chloride channels expressed in the plasma membranes [1,2].

In human medicine, the expression of DOG1 has been accurately evaluated in normal tissues and in a large number of neoplastic tissues by tissue microarray immunohistochemistry. In normal tissues, DOG1 is highly expressed in interstitial cells of Cajal (ICCs) of the gastrointestinal tract, where it plays an important role in mediating intestinal motility. Mast cells, melanocytes, breast ductal epithelium, seminiferous tubules, a subset of glial cells, ovarian stroma, follicles, corpora lutea, and oocytes comprise the cellular substrate in which the immunohistochemical expression of DOG1 is clearly evident [3,4,5,6,7,8,9,10,11,12]. This finding reflects the potential role of this protein in secretory, sensory, and contractile functions [13].

Among human tumors, DOG1 is expressed with high sensitivity and specificity in the majority of gastrointestinal stromal tumors (GISTs) and is actually considered to be the most specific marker for this neoplastic entity. However, studies have shown that DOG1 expression can also occur in various other cancer types, including head and neck squamous cell carcinoma, parathyroid tumors, breast, pancreatic, and gastric carcinomas [11,14,15,16,17,18,19,20,21,22,23,24,25].

Furthermore, in humans, DOG1 appears to play an important role in cancer cell biology. In fact, overexpression of this protein resulted in aggressive tumor cell viability [14,15,16,17,18,26].

In animal tissues, DOG1 expression has been tested by immunohistochemistry (IHC) only in canine GISTs [27,28] and compared to KIT, with conflicting results. While one argues that DOG1 IHC had better specificity than KIT in diagnosing canine GISTs [27], the other one, on the contrary [28], demonstrates that not all GISTs were immunoreactive for DOG1. Furthermore, DOG1 immunoreactivity has not yet been investigated in normal and neoplastic canine tissues.

Focusing on elucidating the role of the DOG1 in dogs, our aim is therefore twofold: (1) to investigate the IHC expression and distribution of the DOG1 protein in both normal and neoplastic canine tissues, using the same antibody previously employed in human studies [29,30] and in canine GISTs [27,28] and (2) to assess the specificity and diagnostic predictive value of DOG1 in a large cohort of canine GISTs.

## 2. Materials and Methods

### 2.1. Samples Collection

A retrospective study on archived formalin-fixed paraffin-embedded (FFPE) samples of canine tissues received for diagnostic purposes at the Pathology Service Department of Veterinary Medical Sciences (DIMEVET) of the University of Bologna (Ozzano Emilia, Bologna, Italy) was conducted. For normal canine tissue, we selected samples fixed in formalin for no more than 24 h from surgical excision of various types of lesions that contained marginal areas of healthy tissue, in a number of 5 different samples for each type of tissue, for a total of 55 samples. With regard to neoplastic tissue, we focused our selection both on a significant number of GISTs (*n* = 24) in our archive and on a significant number of other gastrointestinal mesenchymal neoplasms as well as tumors that expressed KIT in dogs (perivascular wall and mast cell tumors). The samples, 88 in total, came from surgical removals or excisional biopsies and were fixed in formalin for no more than 24 h. We have also selected other individual samples of malignancies on the basis of either significant DOG1 immunoreactivity in the human counterpart or the fact that the cellular counterpart under normal conditions in dogs was strongly DOG1 immunoreactive. The tumors were histologically classified according to WHO criteria on H&E-stained sections, and routine immunohistochemistry (IHC) and/or special stains were applied when necessary to confirm the morphological diagnoses. Mesenchymal intestinal tumors have been classified as gastrointestinal stromal tumors (GISTs) or leiomyoma/leiomyosarcomas based on KIT (CD117) (1:500, polyclonal; cat. no. A4502, Dako, Glostrup, Denmark) expression, with GISTs being KIT immunoreactive and other mesenchymal tumors non-immunoreactive on KIT.

### 2.2. Immunohistochemistry

We performed DOG1 immunohistochemical staining following the streptavidin-biotin-peroxidase technique (BIO SPA, Milan, Italy). The antibodies and their working dilutions were as follows: CD117/c-KIT (1:500, polyclonal; cat. no. A4502, Dako, Glostrup, Denmark) and DOG1 (1:400, rabbit monoclonal, clone SP31; cat. no. 244R-14, Sigma-Aldrich, Darmstadt, Germany). Antigen retrieval was performed in a microwave oven (750 W) for 10 min using citrate buffer (pH 6.0) for CD117 and EDTA buffer (pH 8.0) for DOG1. After 20 min at room temperature, the sections were incubated overnight at 4 °C in a humid chamber overnight with the primary antibody diluted, according to the appropriate dilutions, in 3% BSA (cat. no. A9418, Sigma Aldrich, Darmstadt, DE) and 0.25% Tween20 (cat no. P9416, Sigma Aldrich) in PBS (0.01 M, pH 7.4). The sections, subsequently washed in TRIS buffer, were first incubated with the secondary antibody (anti-rabbit IgG conjugated with biotin, cat no. BA-1000, Vector Laboratories, Burlingame, CA, USA) for 30 min at room temperature, then incubated with the avidin-biotin immunoperoxidase (Vectastain Elite ABC Kit, Vector Laboratories) for 30 min at room temperature. Immunoreactions were detected with the chromogen 3,3′-diaminobenzidine (0.05% *w*/*v*, cat no. ACB999, Histo-Line Laboratories, Pantigliate, Italy). Slides were counterstained with Harris’s hematoxylin (Histo-Line Laboratories). For positive control purposes, we included a section of canine intestinal tissue containing a plexus with interstitial cells of Cajal, which are known to express DOG1 [27]. We performed negative controls by processing tissue sections under identical conditions, except for the omission of the primary antibody, in order to assess non-specific background staining attributable to the detection system. We acquired images with an optical microscope (Eclipse E600; Nikon, Shinjuku, Japan) equipped with the Imaging Source “33” Series USB 3.0 Camera (cat. no. DFK 33UX264; Bremen, Germany).

The immunohistochemical assessment was independently performed by three recognized pathologists who reached a consensus.

The expression was evaluated semi-quantitatively as weak, moderate, or strong. The distribution was scored as negative when <10%, 1 when >10% and <50%, 2 when >50% and <80%, and 3 when >80%. Additionally, the cellular localization (membranous, cytoplasmic, and/or nuclear) was recorded.

Antibody sensitivity for the diagnosis of GISTs was calculated as the proportion of positive samples among mesenchymal intestinal tumors.

## 3. Results

### 3.1. DOG1 in Normal Tissues

DOG1 immunohistochemical expression in normal tissues was tested out of a total of 55 samples. The results obtained, compared with the immunoreactivity on the same cell type in humans using the same clone as in our study, are detailed in Table 1.

**Figure 1:**
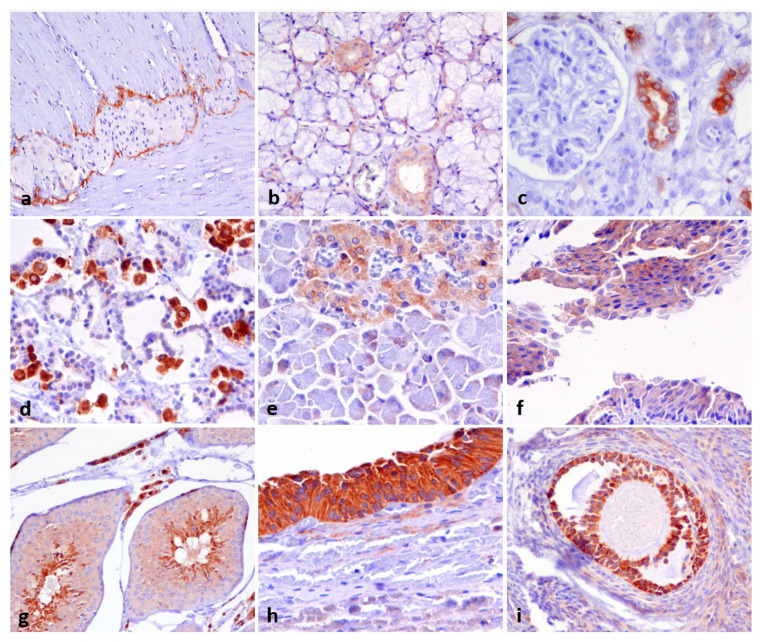
DOG1 immunostaining of normal canine tissues. Panels show DOG1 positivity of (**a**) interstitial cells of Cajal in the intestinal wall (strong and cytoplasmic), (**b**) moderate cytoplasmic staining of the salivary gland duct and apical membranous staining of the acinar cells, (**c**) strong positivity of the renal distal tubule, (**d**) strong cytoplasmic positivity of the parafollicular cells of the thyroid, (**e**) moderate cytoplasmic staining of islet of Langerhans of the pancreas, (**f**) moderate membranous DOG1 staining of the urothelium, (**g**) strong apical positivity of Leydig cells and spermatocytes of the testis and moderate staining of spermatogonia and Sertoli cells, (**h**) strong staining (cytoplasmic and membranous) of ovarian follicle granulosa cells and strong staining of external theca cells, (**i**) strong cytoplasmic staining of oocyte germ cells. Chromogen DAB. Objective: 20× (**a**,**b**,**g**,**i**) and 40× (**c**–**f**,**h**).

DOG1 expression was predominantly cytoplasmic but often accompanied by a membrane pattern (Figure 1). Strong staining was observed in ICCs, external theca cells of the ovary, oocytes, spermatocytes, Leydig cells, renal epithelial cells, and parafollicular cells of the thyroid. We observed weak or moderate cytoplasmic expression of DOG1 in the other cell types listed in Table 1. We also found a moderate apical immunoreactivity in acinar cells of the submandibular salivary gland and a nuclear expression in all Leydig cells of the testis.

### 3.2. DOG1 in Neoplastic Tissues

**Figure 2:**
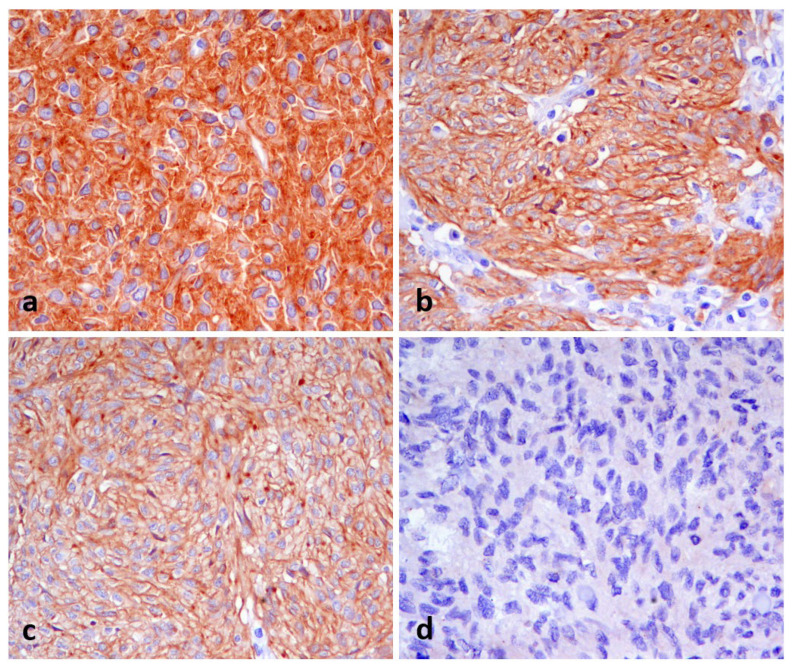
Representative images of DOG1 immunostaining of canine gastrointestinal stromal tumors (GISTs) and leiomyosarcomas of the dog. Panels show (**a**) strong cytoplasmic, (**b**) membranous, and (**c**) membranous with perinuclear aggregates DOG1 immunopositivity of canine GIST and a negative canine intestinal leiomyosarcoma (**d**). Chromogen DAB. Objective: 40×.

DOG1 immunoreactivity has been tested in intestinal mesenchymal tumors on a series of 24 GISTs, 2 leiomyomas, and 8 leiomyosarcomas. A summary of these results is presented in Table 2. All GISTs were immunoreactive for DOG1 except two cases (92%, 22/24), and 1 out of the 8 leiomyosarcomas was found to be immunopositive for DOG1, thus allowing for its reclassification as GISTs. As for GISTs, DOG1 expression varied from weak to strong, and we identified the following three distinct expression patterns: membranous, cytoplasmic, and perinuclear, or combinations of these patterns (Figure 2a–d). As for leiomyosarcoma, DOG1 expression was strongly associated with a cytoplasmic pattern.

Based on the reclassification of DOG1-positive/KIT-negative intestinal mesenchymal tumors as GISTs, the assessment of the diagnostic sensitivity of DOG1 in GISTs is found to be 92% (23/25).

**Figure 3:**
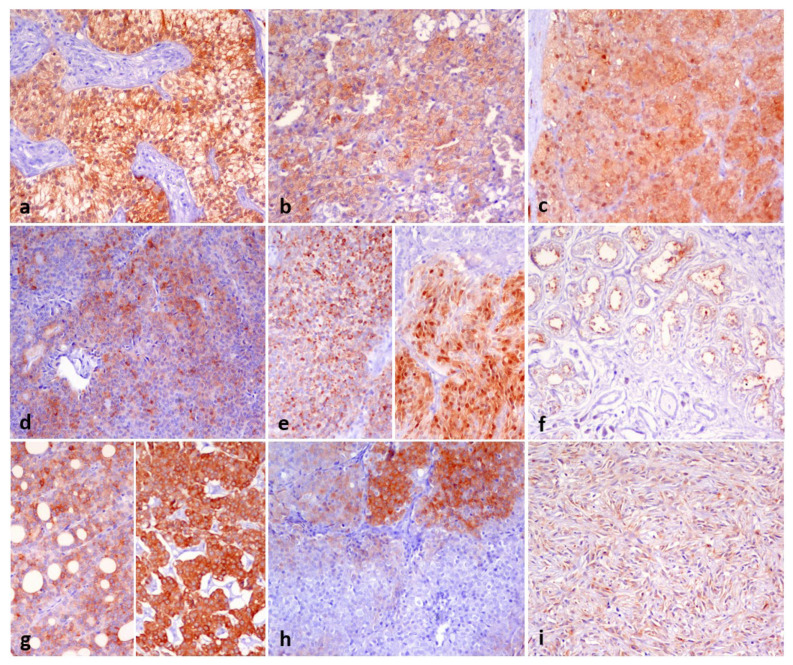
DOG1 immunostaining of canine pathological tissues. Panels show moderate to strong DOG1 positivity of (**a**) Sertoli cell tumor (strong membranous), (**b**) adrenocortical carcinoma (moderate membranous and cytoplasmic), (**c**) adrenal adenoma (moderate membranous and cytoplasmic with strong nuclear positivity), (**d**) vaginal carcinoma (**e**) moderate cytoplasmic with strong perinuclear aggregates staining of granulosa cell tumor of the ovary, (**f**) weak apical staining of sebaceous carcinoma of the skin, (**g**) strong cytoplasmic and membranous staining of two different thyroid carcinomas, (**h**) strong focal staining of solid-follicular carcinoma of the thyroid, (**i**) moderate cytoplasmic to nuclear staining of perivascular wall tumors. Chromogen DAB. Objective: 20×.

Other tumoral entities, such as various types of neoplasms in different locations, were also analyzed. Detailed results, referring to the expression of each specific tumor type in humans using the same clone as in our study, are summarized in Table 2, and some examples of DOG1 immunostaining are shown in Figure 3.

Among the mesenchymal tumors, all the 6 perivascular wall tumors analyzed exhibited positive immunoreactivity, with staining intensities ranging from strong to moderate and constant cytoplasmic and nuclear colocalization.

In epithelial tumors, we observed strong cytoplasmic expression of DOG1, often with a membranous pattern, in one adenocarcinoma of the lung, carcinomas of the thyroid gland (3/4), and one adrenal cortical adenoma. We found weak and moderate cytoplasmic immunoreactivity in one carcinoma of the lung, one thymic carcinoma, one carcinoma of the vagina, one adenoma, one carcinoma of the mammary gland, one urothelial carcinoma, one carcinoma of the kidney, one adrenal cortical adenoma, and one adrenal cortical carcinoma.

With regard to sex-gonadal stromal tumors, we observed strong cytoplasmic expression of DOG1 with also perinuclear aggregates in one granulosa cell tumor. We found weak and moderate cytoplasmic staining in Sertoli cell tumors; conversely, no immunoreactivity was found in Leydig cell tumors.

Finally, we found a moderate cytoplasmic and membranous DOG1 immunoreactivity in 1 out of 6 cutaneous mast cell tumors.

The details of these results are described in Table 2.

## 4. Discussion

DOG1 protein is a plasma membrane protein consisting of 8 transmembrane domains belonging to the family of calcium-activated chloride channels (CaCCs) [1,2]. The main function of the CaCCs is to regulate the efflux of chloride ions, which, in response to intracellular calcium levels, control the normal physiological cell functions, including smooth muscle contraction [34,35]. In normally functioning cells, DOG1 protein is highly expressed in gastrointestinal interstitial cells of Cajal (ICCs) and is involved in regulating ICC proliferation and normal contractility by mediating intestinal motility [36].

Recent IHC investigations on a large number of human tissues, conducted under highly standardized conditions with specific anti-DOG1 antibodies (MSVA-201M and clone SP31) [29], have shown that, in addition to a strong membrane positivity occasionally accompanied by a weak-to-moderate cytoplasmic positivity seen in ICCs, other cells, such as the epithelium of seminal vesicles and serous epithelial cells of salivary glands, also exhibit positivity with similar expression and intensity. Additionally, other cell types, although to a lesser extent both in intensity and cellular distribution, showed positive reactions, including the superficial epithelium of the stomach, gallbladder, colonic goblet cells, fallopian tube epithelium, mammary myoepithelial cells, endometrial and endocervical gland cells, endometrial stroma, and spermatocytes (only with the clone SP31), while all other components were non-immunoreactive.

The explanation for the distribution of DOG1 may lie in its properties as a calcium-activated chloride channel. DOG1 protein mediates transepithelial ion transport and is thus involved in the regulation of airway fluid secretion, the secretory functions of exocrine glands, renal function, vascular smooth muscle contraction, and nociception [1,2].

However, DOG1 owes its discovery and, consequently, its name to a particular histotype of stromal gastrointestinal neoplasia (DOG1 is, in fact, the acronym for ‘Discovered On Gastrointestinal stromal tumor 1′). Specifically, its expression on GISTs was discovered during the analysis of tissue microarray results comparing various sarcomas [37,38].

DOG1 protein is extensively studied and used, in co-expression with KIT, as a marker for diagnosing human GISTs to differentiate from other histomorphologically indistinguishable intestinal spindle tumors, such as leiomyosarcomas. The highest rate of positivity and the highest levels of expression were seen in human GISTs (97.8% to 100% of positive cases), establishing DOG1 as a key diagnostic marker for GISTs [12,29,33,37,39,40,41]. The important role of DOG1 expression in GISTs is further supported by evident associations between high DOG1 expression levels, large tumor size, and unfavorable prognosis [15,42]. Upregulation of DOG1 has been shown to correlate with increased cancer cell aggressiveness [15,16,17], and DOG1 depletion or inhibition has been observed to reduce tumor growth in several cancer models [15,16,43,44].

In dogs, the DOG1 protein has been shown to have a high homology (93% amino-acid sequence identity) with the human protein and has already been tested in a small number of studies dealing with canine GISTs [27,28]. The immunohistochemical and Western blot findings demonstrating specific DOG1 immunoreactivity in interstitial cells of Cajal (ICCs) within normal canine intestinal tissue [27] are in line with established human studies. These data support the interpretation that DOG1-positive gastrointestinal tumors in dogs originate from cells exhibiting ICC differentiation, thereby substantiating a diagnosis of gastrointestinal stromal tumor (GIST). To date, no data is available on DOG1 expression in canine normal tissues or in non-GIST neoplasms.

Using the same monoclonal antibody previously tested in dogs (Clone SP31) on numerous samples representing different tissues under normal conditions, our results appear to be largely comparable to the findings reported in humans. In fact, this allowed us to observe a strong positivity not restricted to ICCs but also present with the same strong expression in numerous cellular components, such as germinal cells (external theca cells of the ovary, oocytes, spermatocytes, and Leydig cells), renal epithelial cells, and parafollicular cells of the thyroid. Regarding the type of DOG1 immunopositivity, in dogs it seems to be more frequently concentrated in the cytoplasm and membrane, while in humans, most positive cellular components showed only membrane positivity, with occasional weak cytoplasmic positivity.

Among the tumor types tested with DOG1, GISTs are the most numerically represented in our case series, selected specifically to analyze the expression and distribution of DOG1 as a starting point for comparisons. Among the few studies using DOG1 in canine GISTs, only Dailey et al. (2015) [27] extensively focus on antibody reactivity and discuss their results in depth. These authors, after confirming similar immunoreactivity to that reported in corresponding human tissues, suggest that DOG1 detection by IHC showed better sensitivity and specificity than KIT for differentiating between GIST and other gastrointestinal spindle cell neoplasms. In fact, in their study, there are no KIT-positive/DOG-negative cases, unlike in our study, in which two cases showed no immunoreactivity to DOG1 despite being undoubtedly positive for KIT. Conversely, one gastrointestinal neoplasm was classified as leiomyosarcoma also on the basis of weak (interpreted as non-specific or artefactual) KIT positivity, also present in three other DOG1-positive GISTs. Under their interpretation, DOG1 demonstrates better sensitivity than KIT for the diagnosis of canine GISTs, a finding consistent with established human data. All DOG1-positive tumors showed reactivity in over 75% of neoplastic cells, including three of the four cases with weak KIT expression. Contrary to this, in our results, although DOG1 positivity was distributed to the vast majority of neoplastic cells, its intensity varied and was not always moderate to strong, unlike the intense and widespread KIT positivity in the same cases. According to our interpretation, DOG1 demonstrates a sensitivity that, although still very high, appears slightly lower than that of KIT for the diagnosis of GISTs (92% vs. 96%, respectively). This interpretation aligns with the results of del Alcázar et al. (2021) [28], where it was reported that, among 32 KIT-positive canine GISTs, only 22 (69%) were also DOG1-positive.

As for DOG1-positive/KIT-negative cases, Dailey et al. (2015) [27] report only one case (as we do, but in a larger cohort), discussing the possibility that these neoplasms represent a distinct GIST subtype derived from a KIT-negative ICC subpopulation or, alternatively, represent cases in which KIT immunostaining yielded false-negative results due to technical variables—such as antigen retrieval issues, epitope masking, or antibody sensitivity—while DOG1 detection remained robust. This second hypothesis is not confirmed in our study. Irrespective of the underlying mechanism, the presence of DOG1 immunoreactivity in GI stromal neoplasms strongly supports the classification as GISTs.

These observations highlight, however, a fundamental diagnostic advantage of integrating DOG1 into immunohistochemical panels for the diagnosis of GISTs, not so much because of its ability to reliably identify GISTs that might otherwise be misclassified or overlooked due to ambiguous or borderline KIT immunoreactivity, but because it enables the detection of GISTs among neoplasms that, due to their histomorphology and KIT negativity, would not otherwise have been recognized.

Our results detect several neoplasms that showed strong, extensive positivity, comparable to that found in human counterparts, including epithelial histotypes. In gastrointestinal neoplasms, spindle-cell tumors that were KIT-negative (10 cases) were all not immunoreactive, except for one case in which strong, cytoplasmic, and diffuse positivity was observed. This partially aligns with what has been observed in humans, where, given the low frequency and low level of expression of DOG1 in non-GIST spindle cell tumors, a high level of DOG1 expression is more indicative of GISTs [12,35,39]. However, unlike in humans, we found that other spindle cell tumors in different locations, such as the skin and perivascular tumors, were DOG1 positive (6/6, 100%), showing moderate to strong positivity, diffuse, and cytoplasmic positivity. Surprisingly, in all cases, this was also associated with strong nuclear positivity, conceivable as a probable aberrant co-location.

Additionally, epithelial neoplasms such as adrenal gland adenoma and carcinoma, thyroid and lung carcinoma, or sex-gonadal endocrine tumors like granulosa cell and Sertoli cell tumor, even if represented as single cases, show moderate to strong DOG1 positivity, distributed across more than 50% of the neoplastic cells. This result highlights the fact that metastases of these neoplasms in the gastrointestinal tract could be misinterpreted as epithelioid histotypes of GISTs. At the same time, it suggests the possibility of cancer cell-associated molecular mechanisms, which could imply a potential effect of channel blockers on disease progression, as already demonstrated in various human tumors [15,16,43,44,45,46].

Indeed, in cancer cells, blockage of DOG1 by chloride channel inhibitor or monoclonal antibodies such as trastuzumab and/or cetuximab appears to reduce cell proliferation and viability, leading to cell cycle arrest or increased rates of apoptosis [15,16,43,44]. For therapeutic applications, antibody–drug conjugates (ADCs) are the most well-established class comprising a cytotoxic agent linked to a targeting ligand that enables site-specific drug delivery to one or more tumor sites. By combining the high specificity of monoclonal antibodies with the potent cytotoxicity of chemotherapeutic agents, ADCs aim to enhance antitumor efficacy while minimizing systemic toxicity [46]. Recent studies [45] suggest that an antibody–drug conjugate targeting DOG1 (as anti-DOG1-DM4-ADC) represents a promising therapeutic approach for DOG1-positive gastrointestinal tumors and may effectively prevent recurrence following curative resection of colorectal liver metastases.

Our study does not address clinical parameters that could support prognostic speculation and is strictly limited to immunohistochemical analysis. Additionally, the absence of molecular data represents a significant limitation in the biological interpretation of the observed expression patterns.

We found that DOG1 is expressed by several normal cellular components in addition to ICCs, and it is confirmed as an adequate immunomarker for GISTs, though it is slightly less specific than KIT. Regarding neoplastic tissues, spindle cell tumors other than GISTs, such as perivascular tumors, showed positivity in most of the cases investigated. In addition, several epithelial tumors displayed moderate to strong DOG1 positivity. Mast cell tumors, typically immunoreactive to KIT, were found to be negative in the vast majority of cases.

The small number of neoplastic entities investigated represents another significant limitation of this research. Although the presence of neoplasms other than GISTs showing similar immunoreactivity has been detected, future research involving larger, more comprehensive case series is necessary to support and further investigate the preliminary findings presented here. Our limited results related to tumors other than GISTs could, however, represent a preliminary contribution for future investigative research into the potential role of DOG1 in tumor oncogenesis.

## 5. Conclusions

The results of our study, in addition to demonstrating the significant expression of DOG1 in numerous normal and neoplastic canine tissues, lead us to conclude that, while KIT remains a highly sensitive marker for canine GISTs in our cohort, DOG1 provides indispensable complementary value by identifying tumors non-immunoreactive for KIT and emphasizes the combined use of both markers in order to increase GISTs diagnostic accuracy.

## Figures and Tables

**Table 1:** DOG1 immunoexpression in healthy tissues of dogs, with the human counterpart. For each organ, the expression of the predominant cell types is indicated. Intensity is expressed as follows: S = strong, M = moderate, and W = weak. Distribution is as follows for each cell type: 3 = >80%, 2 for >50% and <80%, 1 = >10% and <50%, and 0 = r < 10%. Cellular localization is as follows: C = cytoplasmic, N = nuclear, Mb = membranous. NT = not tested.

Normal Tissues	Dog	Human (References)
**Gastrointestinal tract**		
Interstitial cells of Cajal	S, 3, C	Strong [29,31]
Lymphoid-associated tissues	M, 3, C	Negative [29]
**Mammary gland**		
Epithelial cells	W, 2, Mb	Negative [30]
Myoepithelial cells	W, 2, C	Weak [29] Strong [30]
**Ovary**		
Follicle granulosa cells	M, 3, CMb	Negative [29]
External theca cells	S, 3, C	Negative [29]
Oocyte	S, 3, C	Negative [29]
**Uterus**		
Endometrial epithelium	M, 3, C	Positive [29]
**Testis**		
Spermatogonia	W, 3, C	Negative [29]
Spermatocytes	S, 3, C	Strong [29]
Sertoli cells	M, 3, C	Negative [29]
Leydig cells	S, 3, CN	Negative [29]
Epididymis, epithelial cells	M, 2, CMb	Strong [29]
**Kidney**		
Distal tubule cells	S, 3, C	Negative [29]
Collecting duct cells	S, 3, C	Negative [29]
**Adrenal gland**		
Cortical cells	W, 3, C	NT
**Thyroid**		
Parafollicular cells	S, 3, C	Negative [4]
Follicular cells	W, 2, C	Moderate [4,29]
**Pancreas**		
Islet of Langerhans	M, 3, C	Negative [29]
**Bladder**		
Urothelial cells	M, 2, CMb	Weak [29]
**Salivary gland** (submandibular)		
Duct cells	M, 3, C	Weak [29,32]
Acinar cells	M, 3, Mb	Strong [29,32]
Myoepithelial cells	S, 3, C	NT
**Other cells**		
Pericytes	M, 3, C	NT

**Table 2:** DOG1 immunoexpression in neoplastic tissues of dogs, with references to the human counterpart. Intensity is expressed as follows: S = strong, M = moderate, and W = weak. Distribution is as follows: 3 = > 80%, 2 = > 50% and <80%, 1 = > 10% and <50%, and negative = < 10%. Cellular localization is as follows: C = cytoplasmic, N = nuclear, Mb = membranous, and P = paranuclear. NT = not tested.

Neoplastic Tissues	Dog (Cases/Cases Examined)	Human (References)
**Mesenchymal tumors**		
GIST	W, 3, CMb (1/24) W, 3, CP (1/24) W, 3, C (1/24) M, 3, C (2/24) M, 3, Mb (2/24) M, 3, CMb (2/24) M, 3, CMbP (1/24) S, 3, CMb (7/24) S, 3, CP (1/24) S, 3, CMbP (4/24) Negative (2/24)	Strong 89–98% [4,29,31,33]
Leiomyoma (intestine)	Negative (2/2)	NT
Leiomyosarcoma (intestine)	S, 3, C (1/8) Negative (7/8)	Weak 2.9% [29]
Perivascular wall tumor (skin)	M, 2, CN (1/6) S, 3, CN (3/6) M, 3, CN (1/6) S, 1, CN (1/6)	NT
Osteosarcoma	Negative (2/2)	Negative [29]
Hemangiosarcoma (spleen)	Negative (4/4)	NT
**Epithelial tumors**		
Lung adenocarcinoma	M, 2, C (1/3) S, 3, C (1/3) Negative (1/3)	Weak 1.8%, Moderate 0.6% [29]
Thymic carcinoma	M, 1, C (1/1)	Weak 12%, Moderate 4% [29]
Vaginal squamous carcinoma	M, 3, C (1/1)	Weak 10.3%, Moderate 2.9%, Strong 4.4% [29]
Breast adenoma (Papillary)	W, 3, CMb (1/2) Negative (1/2)	Positive ~85% [30]
Breast carcinoma (Papillary)	W, 1, C (1/2) Negative (1/2)	Negative [30] Weak 5%, Moderate 5% [29]
Prostatic carcinoma	Negative (2/2)	Weak 1.2%, Moderate 1.2% [29]
Urothelial carcinoma	M, 3, C (1/2) Negative (1/2)	Weak 10.6%, Moderate 3.2%, Strong 5.2% [29]
Carcinoma of the kidney	Negative (1/2) W, 1, C (1/2)	Negative [29]
Adenocarcinoma of the colon	Negative (1/1)	Weak 21.5%, Moderate 4.6%, Strong 3.8% [29]
Thyroid carcinoma	W, 1, C (1/4) S, 3, CMb (2/4) S, 1, CMb (1/4)	Weak 6.2%, Moderate 4.8% [29]
Adrenal cortical adenoma	W, 1, C (1/2) S, 2, CN (1/2)	Negative [29]
Adrenal cortical carcinoma	W, 1, C (1/2) M, 2, CN (1/2)	Weak 4% [29]
Cholangiocarcinoma	Negative (1/1)	NT
Hepatocellular carcinoma	Negative (1/1)	NT
**Sex Cord-Gonadal Stromal Tumors**		
Granulosa cell tumor	M, 2, CP (1/2) S, 2, CMbP (1/2)	NT
Leydig cell tumor	Negative (2/2)	NT
Sertoli cell tumor	W, 1, C (1/2) M, 2, CMb (1/2)	NT
**Others**		
Seminoma	Negative (2/2)	Negative [29]
Islet cell tumors	Negative (2/2)	Weak 7.7% [29]
Mast cell tumor	Negative (5/6) M, 1, CMb (1/6)	NT

## Data Availability

Archived datasets analyzed are available on request from the authors.

## Abbreviations

The following abbreviations are used in this manuscript:

DOG1	Discovered on GIST1
CaCCS	Calcium-activated chloride channels
ANO1	Anoctamin1
GIST	Gastrointestinal stromal tumors
ICCs	Interstitial cells of Cajal
ADC	Antibody–drug conjugate
FFPE	Formalin-fixed paraffin-embedded
